# Cross-membranes orchestrate compartmentalization and morphogenesis in *Streptomyces*

**DOI:** 10.1038/ncomms11836

**Published:** 2016-06-13

**Authors:** Katherine Celler, Roman I. Koning, Joost Willemse, Abraham J. Koster, Gilles P. van Wezel

**Affiliations:** 1 Department of Molecular Biotechnology, Institute of Biology Leiden, Leiden University, PO Box 9505, 2300 AB Leiden, The Netherlands; 2 Department of Molecular Cell Biology, Leiden University Medical Centre, PO Box 9600, 2300 RC Leiden, The Netherlands

## Abstract

Far from being simple unicellular entities, bacteria have complex social behaviour and organization, living in large populations, and some even as coherent, multicellular entities. The filamentous streptomycetes epitomize such multicellularity, growing as a syncytial mycelium with physiologically distinct hyphal compartments separated by infrequent cross-walls. The viability of mutants devoid of cell division, which can be propagated from fragments, suggests the presence of a different form of compartmentalization in the mycelium. Here we show that complex membranes, visualized by cryo-correlative light microscopy and electron tomography, fulfil this role. Membranes form small assemblies between the cell wall and cytoplasmic membrane, or, as evidenced by FRAP experiments, large protein-impermeable cross-membrane structures, which compartmentalize the multinucleoid mycelium. All areas containing cross-membrane structures are nucleoid-restricted zones, suggesting that the membrane assemblies may also act to protect nucleoids from cell-wall restructuring events. Our work reveals a novel mechanism of controlling compartmentalization and development in multicellular bacteria.

## Abstract (web summary)

Streptomycetes are multicellular bacteria that grow as multinucleoid filaments with infrequent cross-walls. Here, the authors describe a membrane system that forms protein-impermeable barriers and compartmentalizes the multinucleoid filaments independently from the FtsZ-guided cell division machinery.

## Graphical abstract

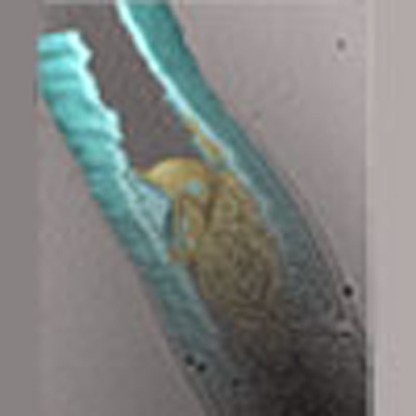

Bacteria have a complex organization on both a unicellular and multicellular level, with many species demonstrating multicellular traits or behaviours analogous to those seen in eukaryotic systems^1^. This multicellularity can manifest itself in different ways, from intercellular communication between individual cells in a population, to a lifecycle spent as a coherent group of cells, such as in biofilms, fruiting bodies, filaments or mycelia^2^,^3^. A case in point are the streptomycetes, filamentous bacteria, which grow forming highly structured multicellular colonies composed of physiologically distinct hyphae^4^,^5^.

The streptomycetes are soil-dwelling saprophytes, well-known for their complex secondary metabolism and ability to produce an abundance of natural products including antibiotics, anticancer agents and immunosuppressants^6^. After a spore has germinated, a complex network is formed as the hyphae grow by tip extension and branching into a syncytial vegetative mycelium separated by occasional peptidoglycan-based septa, with a spacing of some 5–10 μm (ref. 7). Upon nutrient depletion, the streptomycetes undergo extensive chemical and physiological differentiation, with the coordinated production of aerial hyphae and antibiotics^8^,^9^. At this stage in the life cycle, programmed cell death (PCD) occurs in certain parts of the vegetative mycelium and not others, which may be interpreted as a strategy to provide nutrients to the developing aerial mycelium^10^. The aerial hyphae eventually metamorphose into chains of unigenomic spores.

Though cell division is the hallmark of life, in *Streptomyces* it is non-essential and the gene encoding the cell division scaffold protein FtsZ can be deleted^11^. Surprisingly, although it no longer undergoes reproductive cell division and fails to form the cross-walls that are needed to compartmentalize vegetative hyphae, the *ftsZ*-deletion mutant can be propagated from fragments obtained by mechanical maceration of a large syncytial mycelium^11^,^12^. It is a mystery why in the absence of any physical barrier the hyphae do not ‘bleed' to death from open hyphal ends. This strongly suggests that a yet unknown form of compartmentalization of the hyphae exists^13^. Compartmentalization is also implied by the infrequent nature of cross-wall division in the vegetative hyphae, yet prevalence of short live and dead portions of hyphae undergoing differentiation and PCD^14^.

The streptomycetes grow by hyphal extension at apical sites, with highly dynamic cell wall construction and remodelling^15^,^16^. In the absence of a clear mid-cell reference point, growth and division in these multicellular bacteria pose an interesting problem: how can these processes take place without damaging the many chromosomes in the multinucleoid hyphae? In addition, during sporulation-specific cell division up to a hundred septa are formed in the sporogenic aerial hyphae, visualized as spectacular Z ladders over nucleoids that have not yet segregated^17^,^18^,^19^. The canonical DNA-damage control systems such as the SOS response or nucleoid occlusion, which mediate division control in cells with a planktonic lifestyle^20^,^21^,^22^, have not been identified in *Streptomyces.* An alternative system must therefore exist to prevent lethal DNA damage by the cell-wall synthetic machineries.

In this work, we demonstrate the presence of a membrane system in the vegetative hyphae of *Streptomyces*, existing apart from the peptidoglycan-based cell compartmentalization mediated by the FtsZ-guided cell division machinery. These so-called ‘cross-membranes' form protein impermeable barriers between hyphal segments and effectively compartmentalize the multinucleoid hyphae.

## Results

### Intracellular membranes exist in *Streptomyces* vegetative hyphae

**Figure 1:**
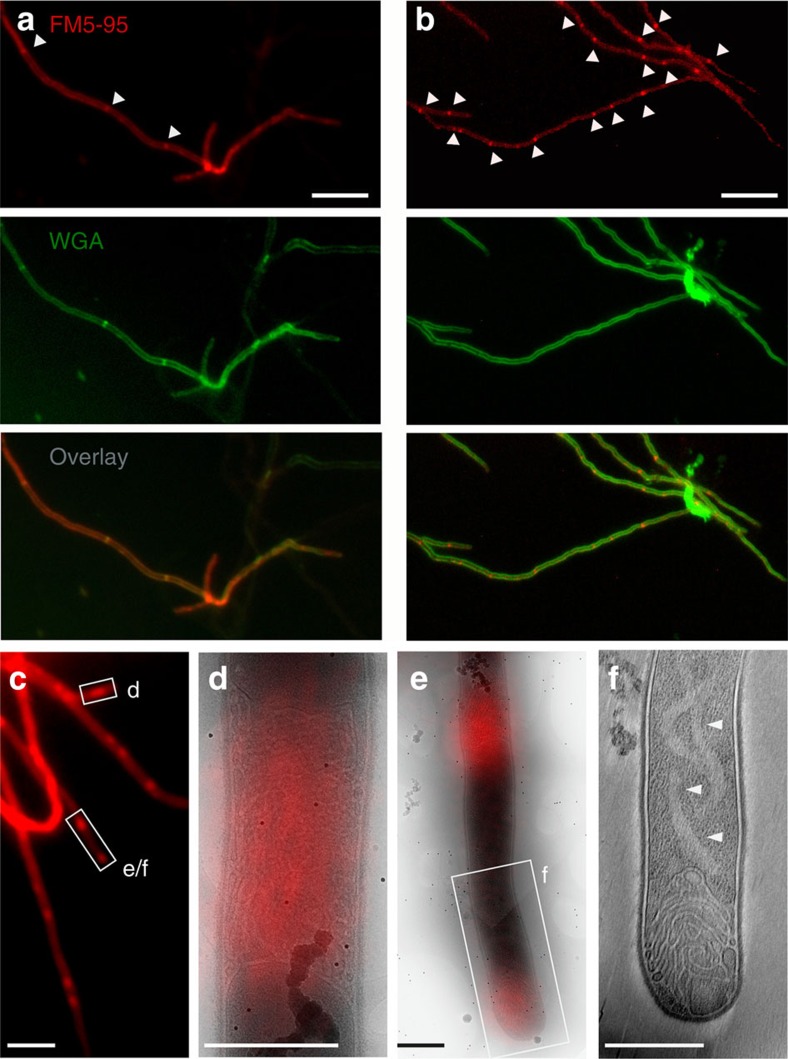
Membrane assemblies in vegetative hyphae. (**a**) In vegetative hyphae of *S. coelicolor* M145 stained with the membrane dye FM5–95 (red) and the cell wall dye FITC-WGA (green), cross-walls are evident (arrowheads). (**b**) Large membrane structures/agglomerates are also found within hyphae, which do not co-localize with WGA-stained cross-walls (arrowheads). (**c**–**f**) To investigate their ultrastructure, membranes in *S. albus* were fluorescently labelled with FM5–95 and imaged with cryo-CLEM. Positions with extended lipids were observed (**d**–**f**). Cross-membrane assemblies within hyphae (**d**,**e**) and at tips (**e**,**f**) consisted of extended tubular membrane structures. A light region, devoid of ribosomes and other macromolecular complexes, can be seen forming a faint ribbon behind a membrane-filled tip (arrows, **f**; for tomogram see Supplementary Movie 1). Scale bars, 5 μm (**a**–**c**), 500 nm (**d**–**f**).

To investigate membrane remodelling in the *Streptomyces* vegetative mycelium, anionic phospholipids including phosphatidylglycerol (P_GL_), were stained with the membrane dye FM5–95, cross-walls stained with FITC-WGA (FITC-wheat germ agglutinin, or alternatively, BODIPY-vancomycin, which binds to the terminal d-Ala within the wall peptide of peptidoglycan^23^) and their localization studied with fluorescence light microscopy (fLM). This not only revealed membrane structures associated with peptidoglycan cross-walls (Fig. 1a), but also in surprisingly large assemblies, several micrometres in length, spanning the width of vegetative hyphae (Fig. 1b). Lipid assemblies were present within hyphae in differing amounts, with some hyphae being devoid of membranes and others containing multiple formations, from small blebs to large structures. Quantification experiments indicated that of 218 membrane localizations counted, 75.3% localized in large or small apparently non-septal assemblies. The remaining 24.7% were at septal locations—either clearly co-localizing with BODIPY-vancomycin or in the absence of BODIPY-vancomycin—still appearing in thin, cross-wall-like form (Supplementary Table 1 and Supplementary Figs 5–8).

We applied cryo-correlative light and electron microscopy (cryo-CLEM), combining cryo-fLM with cryo-electron tomography (cryo-ET), to study the fine structural detail of the membrane assemblies in three dimensions at nanometre resolution. Cryo-CLEM enables direct fluorescent labelling and targeting of molecules or molecular assemblies (such as intracellular membranes, DNA or cyto-structural elements) in the same cryo-immobilized samples that are then visualized by cryo-ET, avoiding the possible artefacts of chemical fixation, plastic embedding and/or metal staining. In this manner, sites of interest can be quickly located without imposing cryogenic samples to extended electron beam damage. We used *Streptomyces albus* in our cryo-CLEM experiments as this strain is morphologically very similar to model organism *S. coelicolor* on agar plates, with abundant sporulation, yet has thinner hyphae in submerged cultures and therefore allows for high-resolution three-dimensional imaging by whole-cell cryo-ET. By performing cryo-CLEM on *S. albus* hyphae stained with FM5–95, we were able to readily target membrane assemblies within hyphae (Fig. 1c). In young vegetative hyphae (12 h after inoculation), membranes could be seen forming bundles of tube-like structures varying from small assemblies along the cell wall to large structures completely delimiting the hyphae (Fig. 1d and Supplementary Fig. 1). The membranes were also identified at apical sites (Fig. 1e,f and Supplementary Movie 1), as seen in fLM of *S. coelicolor*^24^. Similar membrane assemblies were also found in *S. coelicolor* when examined by cryo-CLEM (Supplementary Fig. 2).

**Figure 2:**
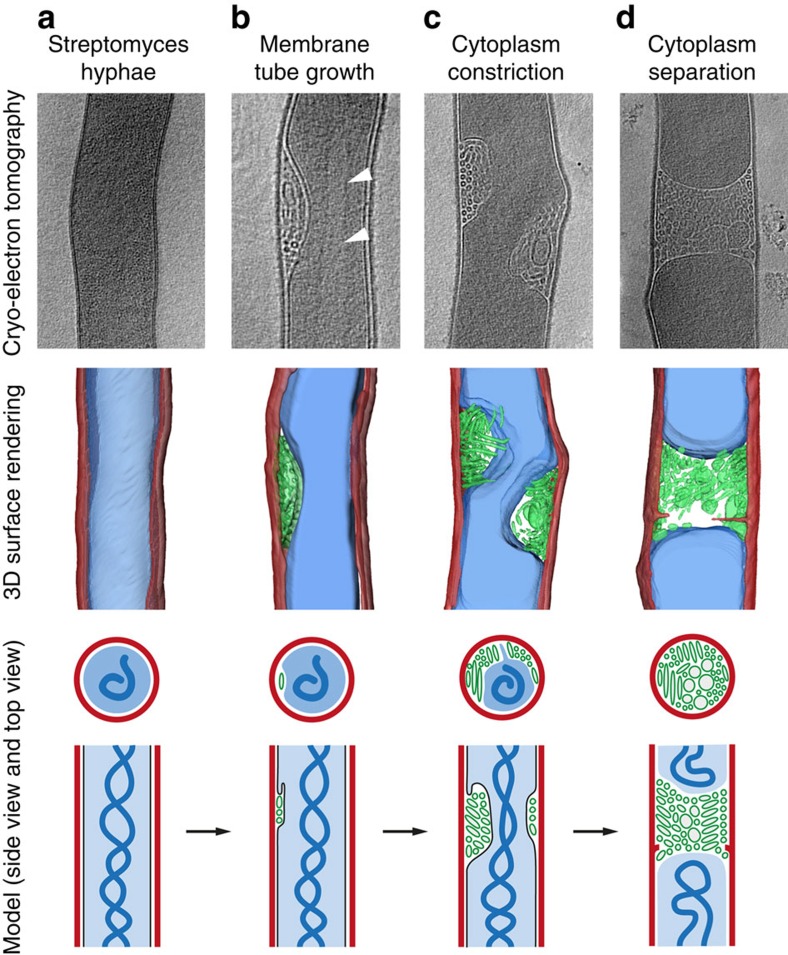
Electron tomography and data segmentation of membrane formations in three dimensions. Cryo-ET slices (top row), surface renderings of the tomograms (middle row) and 2D model (bottom rows, side view and top view) of intracellular membranes in vegetative hyphae of *Streptomyces*. When no membranes are observed, hyphae appear as in **a**. An example of small patches, or blebs, of densely packed lipid tubes between the cytoplasmic membrane and cell wall is given in **b**. White arrowheads denote lighter areas, devoid of ribosomes. In **c**, lipid tubes can be seen increasingly constricting the cytoplasm, whereas in **d**, full cross-membranes are evident, forming a plug across the hypha. In the 2D model, cell wall (red), DNA (dark blue), cytoplasm (light blue), cytoplasmic membrane (black), membrane tubes and vesicles (green) are schematically depicted from orthogonal (third row) and parallel views (fourth row).

Cryo-ET and data segmentation enabled visualization and analysis of the membrane formations in three dimensions, and demonstrated that the assemblies form in the space between the cytoplasmic membrane and cell wall (Fig. 2). Membranes were observed to vary from several small tubes to larger structures, which appeared to be constricting, decreasing the connection between hyphal compartments, likely in an intermediate stage of membrane closure (Fig. 2c). Evidence of septum synthesis could also be seen associated with cross-membranes, and mostly with full cross-membrane structures, which spanned the entire hyphae (Fig. 2d). Tomograms and full 3D surface renderings can be found in Supplementary Movies 2–5.

### Cross-membranes create a chromosome-free area

**Figure 3:**
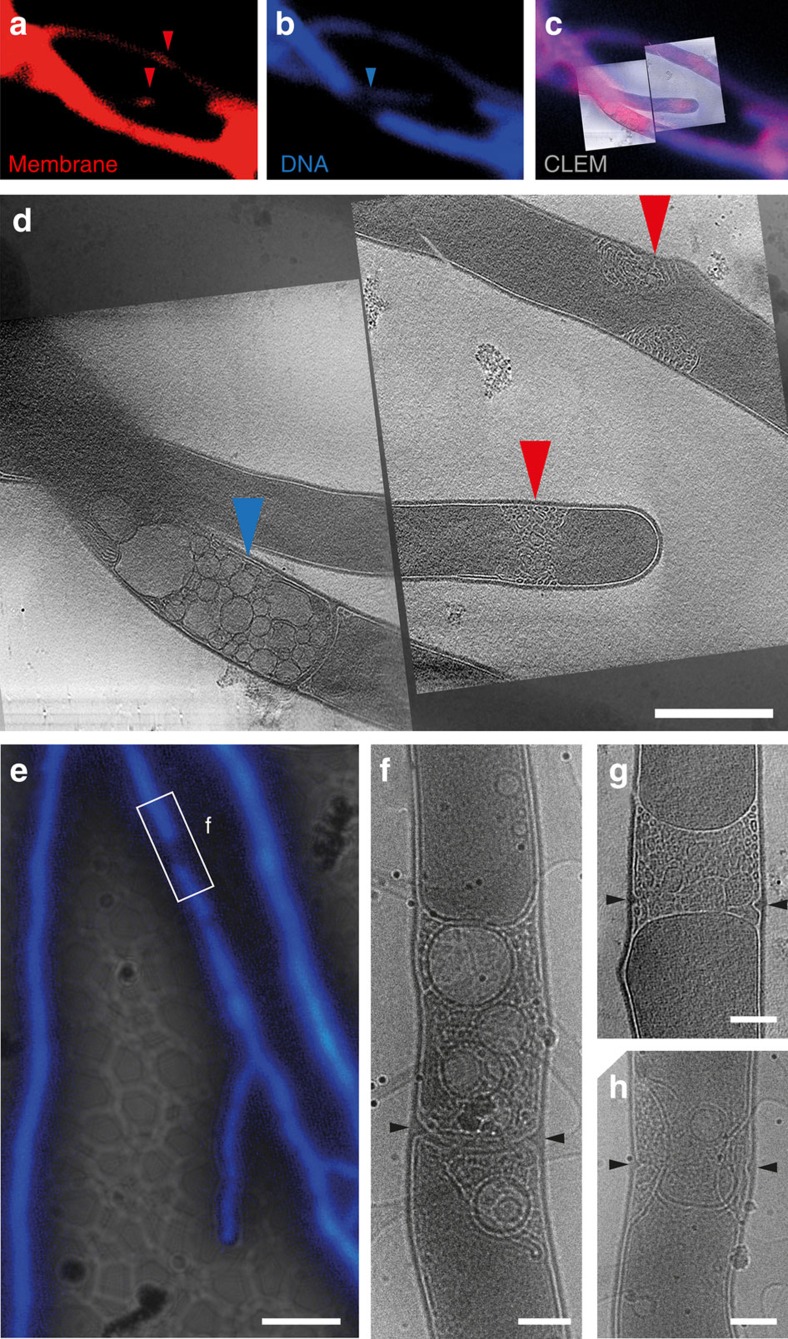
DNA and cross-membranes are mutually exclusive in vegetative hyphae. Vegetative hyphae of *S. albus* fluorescently labelled with FM5–95 for membranes (**a**) and DAPI for DNA (**b**) were imaged by cryo-CLEM (**c**) and cryo-ET (**d**). All areas that contained membrane assemblies (red arrowheads, **a**,**d**) were devoid of DNA. Vesicularization could be seen near a completed cross-wall in an area devoid of DNA (blue arrowheads, **b**,**d**). In a second example, fluorescent labelling of vegetative hyphae of *S. albus* with DAPI (**e**) again revealed positions devoid of DNA (white rectangle denoted **f**) and filled with membranes and vesicles as shown by cryo-EM. Cell wall formation can be seen within the membranes (black arrowheads, **f**). In **g**,**h**, other examples of newly forming cell wall within membranes are shown. Scale bars, 500 nm **a**–**d**; **e**, 5 μm; **f**–**h**, 200 nm.

Excited by the novel visualization of cross-membranes that cryo-ET afforded and to specifically determine the spatial localization of the membranes relative to chromosomes, we combined membrane staining by FM5–95 with chromosome visualization using 4′,6-diamidino-2-phenylindole (DAPI; Fig. 3a–c). It was previously demonstrated that nucleoids are absent from areas enriched in anionic phospholipids^24^. We observed that all areas that showed a distinct gap in DAPI fluorescence in cryo-fLM, and therefore were devoid of DNA, correlated to large hyphae-spanning membrane assemblies (red arrows, Fig. 3a,d) or vesicle structures (blue arrows, Fig. 3b,d). Again, we observed evidence of septum synthesis within full cross-membrane structures, which spanned the entire hyphae (Fig. 3e–h). This correlation, as well as the frequent presence of membranes at hyphal tips, suggested that in *Streptomyces* vegetative hyphae membrane assemblies may act to occlude chromosomal DNA from sites of active cell wall restructuring, either for septum synthesis (Fig. 3f–h), tip growth or branching.

### Membranes form independent of cell wall remodelling

**Figure 4:**
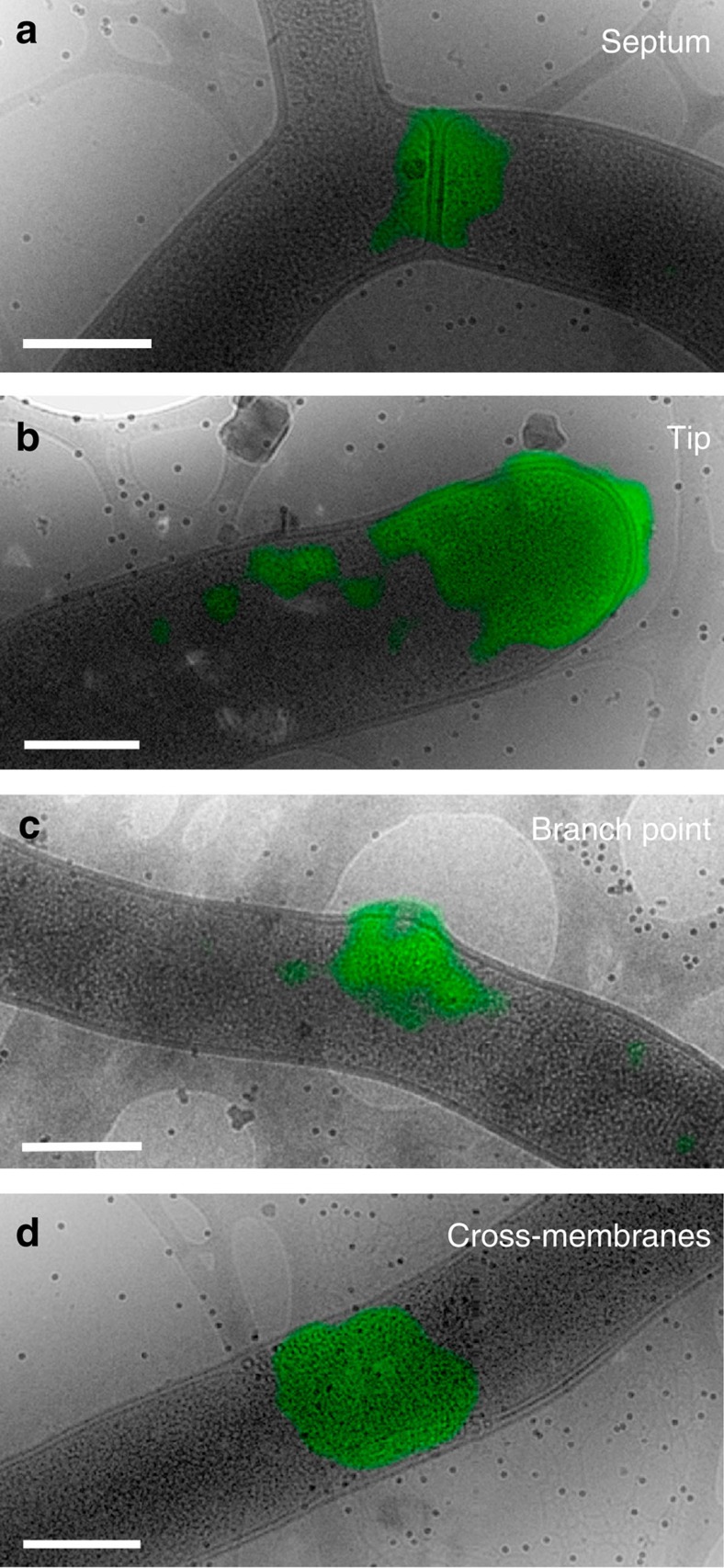
Vancomycin-BODIPY staining shows positions of cell wall restructuring. Cryo-CLEM on vegetative hyphae of *S. albus* that were fluorescently labelled with vancomycin-BODIPY showed that restructuring of cell wall mainly occurs at sites of septa (**a**), but also at tips (**b**), branch points (**c**) and cross-membranes (**d**).

To investigate the relationship between membrane formation and cell wall remodelling, we stained samples for cryo-CLEM with BODIPY-vancomycin and looked for membrane assemblies at locations of BODIPY-vancomycin staining. Experiments revealed numerous examples of BODIPY-vancomycin staining present at septa (Fig. 4a), and to a lesser extent at growing tips (Fig. 4b), and new branch points (Fig. 4c). High-resolution transmission electron micrographs confirmed that some regions stained with BODIPY-vancomycin also contained cross-membranes (Fig. 4d). A statistical analysis on fLM images co-stained with BODIPY-vancomycin and FM5–95 indicated that of a total of 335 locations measured (containing FM5–95 staining, BODIPY-vancomycin staining or both), 117 (34.9%) demonstrated only BODIPY-vancomycin staining (and no FM5–95 staining) at septa or points of cell wall remodelling. Of the remaining locations, 153 (45.6%) demonstrated only FM5–95 staining, and 65 (19.4%) demonstrated both (Supplementary Table 1 and Supplementary Figs 5–8). This result suggests that membranes are either not essential for new cell wall formation, or that if membranes do co-localize with newly forming cell wall, the co-localization is transient. To obtain insight into the dynamics of co-localization during peptidoglycan synthesis, we therefore performed live imaging experiments. These experiments, however, repeatedly failed due to the toxicity of the immunofluorescence stains to young vegetative hyphae.

*Streptomyces* grow by hyphal tip extension, with polar growth and branching depending on DivIVA^25^. As we observed many membrane-filled tips during cryo-CLEM experiments, we were interested to see if these membranes mark the location of DivIVA. We performed fLM studies to quantify the co-localization of this morphoprotein with membranes and found that 27.0% of DivIVA foci co-localize with membrane assemblies (Supplementary Table 2 and Supplementary Fig. 9).

**Figure 5:**
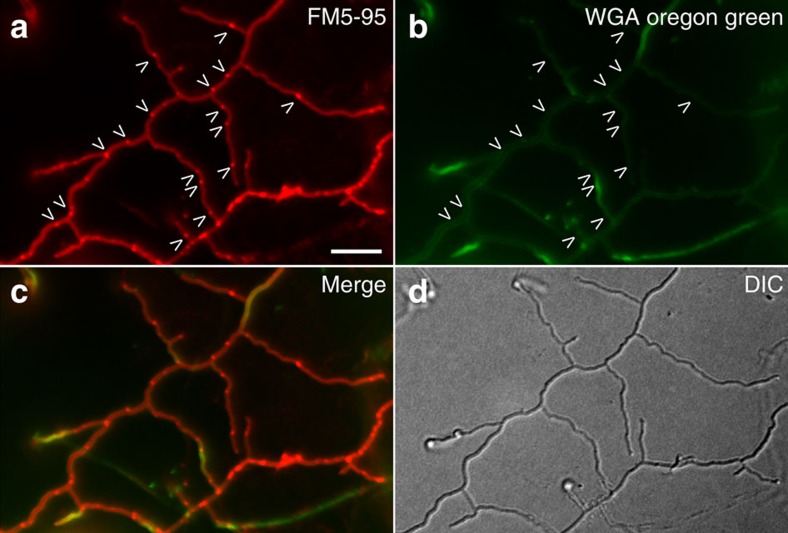
Membrane and cell wall localization in an *S. coelicolor ftsZ*-deletion strain. Membrane assemblies are still abundant in *S. coelicolor ftsZ*-deletion strain (arrows, **a**, stained with FM5–95), but no cross-walls form (arrows, **b**, note *N*-acetylglucosaminyl residues present in cell wall stained with WGA-Oregon green). An overlay image is provided (**c**), as well as the light image (**d**). Scale bar, 5 μm. DIC, differential interference contrast.

Given the incomplete spatio-temporal co-localization of membranes with newly forming cell wall at cross-walls or hyphal tips, we wondered whether cross-membranes form independent of septum synthesis. We therefore quantified membrane and cell wall formation in the *S. coelicolor ftsZ* deletion strain, which grows without forming septa^11^. fLM experiments staining the hyphae with FM5–95 for membranes and WGA for newly forming cell wall demonstrated that in 77 hyphae, with a total length of roughly 2,635 μm, 368 cross-membranes were present and no cross-walls (Fig. 5). This amounts to one cross-membrane per 7.16±0.66 μm. In wild type, the number of cross-membranes is much higher at one cross-membrane per 2.86±0.50 μm (*N*=313 membranes counted), whereas the number of cross-walls formed is one per 9.03±0.46 μm (*N*=99 cross-walls counted). Cryo-electron microscopy (EM) experiments confirmed that the cross-membranes in the *ftrsZ* deletion strain appear indistinguishable to those in wild-type *S. coelicolor* (Supplementary Fig. 3). The complete absence of cross-walls and abundance of cross-membranes in the *ftsZ* deletion strain indicates that septum synthesis is not required for the formation of—and that septa are not necessarily present in—cross-membranes.

### Cross-membranes compartmentalize hyphae

**Figure 6:**
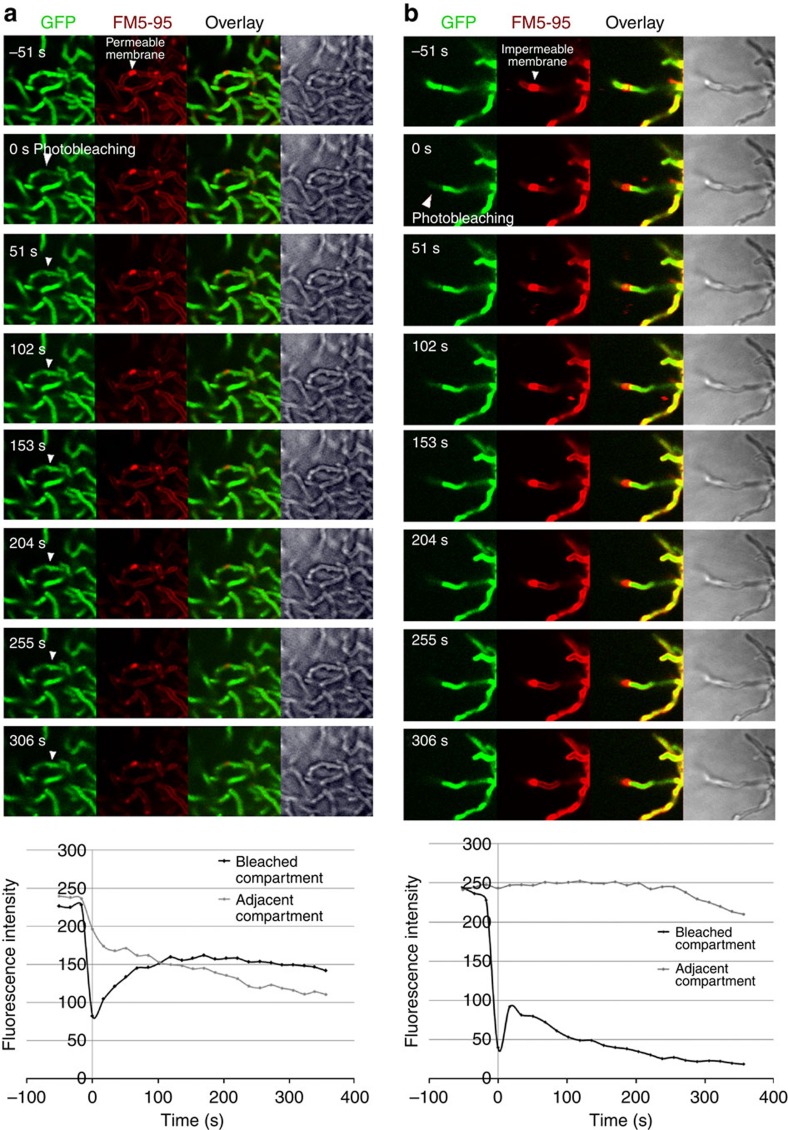
FRAP analysis of GFP distribution over cross-membranes. Fluorescence recovery after photobleaching (FRAP) on a *S. coelicolor* strain constitutively expressing eGFP and stained with FM5–95 for membranes. An entire compartment was bleached on one side of a cross-membrane to establish whether GFP molecules from the adjacent compartment would restore fluorescence. Membranes can be both cytosol permeable (**a** and Supplementary Movie 6), which can be seen when bleached GFP molecules spread through the membrane as indicated by the white arrows, or impermeable (**b** and Supplementary Movie 7), which can be seen when the membrane prevents the flow of bleached GFP molecules. Recovery curves demonstrate the recovery in fluorescence intensity in the case of the permeable membrane, together with a decrease in fluorescence in the adjacent compartment. In the case of the impermeable membrane, a slight increase in fluorescence is seen in the bleached compartment due to diffusion of GFP molecules from within the compartment, whereas there is no decrease in fluorescence in the adjacent compartment. Further imaging results in bleaching and a decrease in fluorescence in both the bleached and adjacent compartments.

Staining of *Streptomyces* mycelium with propidium iodide and SYTO 9 demonstrates alternating live and dead compartments in young vegetative mycelium, suggesting that the PCD linked to development already occurs in very young hyphae^26^. We wondered if the large cross-membrane structures we observe (and not septa) form the cytosol impermeable barrier that enables this type of differentiation. We therefore applied fluorescence recovery after photobleaching (FRAP) on a *S. coelicolor* strain constitutively expressing enhanced green fluorescent protein (eGFP) and stained with FM5–95 for membranes, bleaching an entire compartment on one side of a cross-membrane to establish whether green fluorescent protein (GFP) molecules from the adjacent compartment can restore fluorescence. FRAP experiments (*N*=91) were performed on regions in close proximity to membranes. These experiments demonstrated that in 71% of cases cross-membranes are cytosol permeable (Fig. 6a and Supplementary Movie 6) and in 29% of cases impermeable (Fig. 6b and Supplementary Movie 7), preventing fluorescent GFP migration into the adjacent compartment. Additional FRAP experiments were performed to distinguish cross-membranes containing cross-walls from those that do not contain any cell wall material. In these experiments, the *S. coelicolor* strain constitutively expressing eGFP was stained with both FM5–95 for membranes and far-red dye WGA-Alexa 633 to stain cross-walls. Membranes with no cell wall present were targeted to start. In these cases, cross-membranes were found to be permeable in 23% of cases (green arrows, Supplementary Fig. 4, Supplementary Movie 8) or impermeable in 77% of cases (red arrows, Supplementary Fig. 4, Supplementary Movie 8), revealing that cross-membranes alone can block diffusion within hyphae. Next, membranes with cell-wall material present were targeted. For these cross-membranes, the permeable fraction was 71%, whereas large membrane assemblies near a cross-wall completely blocked diffusion. This increased permeability of cross-membranes with cell-wall material present can be explained by the fact that cross-walls are likely permeable^13^; once cross-membranes disassemble, transport can occur over cross-walls. Taken together, our experiments show that in the absence of cross-walls, cross-membranes can act as impermeable physical barriers between adjacent compartments.

## Discussion

In this work, we discovered the presence of a vast internal membrane system in *Streptomyces* species that can be found within and throughout vegetative hyphae, forming bundles of tubular structures between the cytoplasmic membrane and cell wall. Using cryo-CLEM, we observed membrane formations in small blebs along the cell wall or at hyphal tips, or as large assemblies and hyphae-delimiting structures we termed cross-membranes. Similar structures were observed in the 1960s (refs 27, 28), although their function was unknown. Our present investigation of these membranes resolves several intriguing questions surrounding their role in the *Streptomyces* lifecycle. Although the production of antibiotics and other clinically important secondary metabolites by the streptomycetes has been linked to PCD in the vegetative mycelium, to date, little was known about how the hyphae separate into live and ‘dead' segments.

Peptidoglycan-based septa, or cross-walls, are infrequent in the vegetative hyphae. As beautifully illustrated by live imaging experiments, during hyphal growth of *S. coelicolor* cross-walls (that is, vegetative septa) are produced seemingly randomly at sites distal from the growing tips, delimiting the hyphae into compartments of 5–10 μm (ref. 29). However, the compartments can be up to 40 μm for some other streptomycetes^30^, and these compartments contain approximately one chromosome per micrometre, or tens of copies of the genome per compartment. Cross-walls do not form in the *ftsZ* deletion strain, which raises the question as to how a colony can ensure its survival during PCD or following physical damage to the mycelium. It is now clear that membranes play an important role, offering a less energy-intensive compartmentalization solution than cross-wall formation.

Interestingly, although compartmentalization is rare in bacteria, it does exist in a variety of species^31^,^32^,^33^. Recently, a protein-mediated diffusion barrier was also discovered in the polar stalk of *Caulobacter crescentus*, acting to prevent exchange of membrane and soluble proteins between the stalk and cell body^34^. The cross-membranes in *Streptomyces* similarly delimit and prevent diffusion of soluble proteins between diverse hyphal segments, as evidenced by FRAP experiments on the diffusion of molecules of GFP.

Our FRAP experiments revealed that 29% of cross-membranes are impermeable. Although unlikely, it is conceivable that laser-mediated damage to the cross-membranes may have contributed to leakage of GFP from adjacent compartments, increasing the number of permeable membranes we counted. The percentage of impermeable cross-membrane barriers may therefore be higher than what we observed. Molecules smaller than GFP may also be able to pass the cross-membranes. The high percentage of permeable membrane assemblies we observed are likely membrane structures that are not yet fully closed, that is, in an earlier stage of cross-membrane formation, or perhaps these membranes serve a different purpose during the *Streptomyces* lifecycle.

Indeed, the abundance of lipid structures localizing in small blebs adjacent to the cell wall, in addition to the full cross-membranes delimiting hyphae, could indicate that smaller membrane assemblies may be involved in cellular functions other than compartmentalization. At locations along the hyphae, small blebs are likely mainly precursors for cross-membranes. However, membrane assemblies are also frequently observed at hyphal tips, which suggests that they may be involved in tip growth. It follows that some of the membrane blebs along the hyphae may not be precursors for cross-membranes, but rather the future sites of branching. At growing tips and new branches, it is likely that the assemblies observed have a function in creating a nucleoid-free zone in a region of high activity.

In the *Streptomyces* growing apex, a highly dynamic tip-organizing complex (the so-called TIPOC) exists, consisting of many components—including various cytoskeletal elements—and ensuring that DNA replication takes place at some distance behind the tip^16^,^35^,^36^. Extensive statistics using cryo-ET are not feasible considering the high-resolution nature of these experiments, so instead we performed static fLM experiments on vegetative hyphae stained with FM5–95 and peptidoglycan marker BODIPY-vancomycin and tip growth protein DivIVA to determine the degree of membrane co-localization with new cell wall synthesis. These experiments (see Supplementary Tables 1 and 2 for details) did not indicate complete spatio-temporal co-localization. It should be noted, however, that studying dynamic events with static experiments is notoriously difficult. Further experiments using other cell division or morphoproteins can perhaps provide more insight into the role of the membranes in providing a suitable environment for protein localization. In addition, use of an integrated system for live light microscopy and cryo-fixation (for example, the Rapid Transfer System^37^ or the MAVIS^38^) could enable the targeting of actively growing tips or cross-walls at different stages of formation, and elucidating the role of membranes therein.

Finally, although we demonstrated that cross-membranes form independent of septum synthesis, during our experiments, we frequently observed cross-membranes containing emerging cross-walls (Fig. 3f–h). In the multicellular streptomycetes, hyphal compartments are multigenomic; it is likely that some form of subcellular organization exists that serves to separate the chromosomes. One obvious reason for such compartmentalization would be to allow cross-walls to form in a chromosome-free area.

In conclusion, we show here that cross-membranes exist in the hyphae of the streptomycetes, acting to compartmentalize the hyphae. These membranes can be cytosol permeable or impermeable, and provide a plausible answer to the question why hyphae of the *ftsZ* null mutant do not ‘bleed' to death but rather can be fragmented and propagated. Further, the cross-membranes create DNA-free zones and offer a solution to the long-standing problem of how cell wall remodelling can take place without DNA damage in the multinucleoid hyphae. Future technological advances in correlative microscopy, in which fluorescently tagged proteins can be superimposed on high-resolution images of cytostructural elements in tomograms, should provide further insights into the exciting cell biology of the streptomycetes.

## Methods

### Bacterial strains and constructs

*S. coelicolor* and *S. albus* strains were grown on R2YE agar or soya flour mannitol agar. Liquid cultures were grown in 2YT medium supplemented with 10% sucrose. All of the growth medium recipes used are those mentioned in the ref. 39. For fluorescence microscopy, samples from liquid cultures were spotted onto a glass microscope slide before microscopy analysis. Images of vegetative hyphae from solid growth samples were collected from samples that had been inoculated at the acute-angle junction of coverslips inserted at a 45° angle in soya flour mannitol agar plates. Strains used in this study include *S. coelicolor* M145 (ref. 39), obtained from the John Innes Centre strain collection, its derivative K202 containing plasmid KF41 that expresses FtsZ-GFP^17^, *S. albus* subspecies *albus* G. ATCC 25426 and, for FRAP, a *S. coelicolor* strain constitutively expressing GFP. This strain was created by transforming *S. coelicolor* M145 with pGreen, a pIJ8630 derivative harbouring the constitutive *gap1* promoter of *S. coelicolor* A3(2) M145 cloned upstream of the *eGFP* gene (B. Zacchetti and D. Claessen, unpublished observations).

### Microscopy

*Fluorescence microscopy*. Fluorescence and corresponding light micrographs were obtained with a Zeiss Axioscope A1 upright fluorescence microscope (with an Axiocam Mrc5 camera at a resolution of 37.5 nm per pixel), with, for the green channel, 470- to 490-nm excitation and 515 long-pass detection; and for the red channel, 530- to 550-nm excitation and 590 long-pass detection. For cryo-stage imaging, the green fluorescent images were created using 470/40-nm band-pass excitation and 525/50 band-pass detection; for the red channel, 550/25-nm band-pass excitation and 605/70 band-pass detection were used. For staining of the cell wall (peptidoglycan), we used FITC-WGA; for membrane staining, we used FM5–95 (both obtained from Molecular Probes). FRAP experiments were carried out on a Zeiss Imager system, using 488 nm excitation for eGFP with 505–530 detection and 543 nm excitation for FM5 27,28(refs 27,28) 5–95 with LP 560 detection. Bleaching was performed with 25% laser intensity for 8 s. Images were recorded every 17 s and post-bleaching was monitored for 5 min. All images were background-corrected, setting the signal outside the hyphae to 0 to obtain a sufficiently dark background. These corrections were made using Adobe Photoshop CS5. Triple coloured FRAP experiments were performed with 488 nm excitation, which does not excite WGA-Alexa 633 and therefore the same band-passes were used for signal detection: for example, for eGFP, band-pass filter 525/50; for FM5–95, long-pass 560. WGA-Alexa 633 was imaged with 633 excitation and long-pass 650 detection.

*Sample preparation for electron microscopy*. A small drop (3 μl) of *Streptomyces* liquid culture was applied to EM grids and vitrified by plunging into a liquid ethane/propane mixture using a vitrobot Mark IV (FEI Company) operated at 22 °C and 100% humidity using 1–2 s blotting. Plunge-frozen grids were stored in liquid nitrogen until further use. For cryo-electron tomography, 15 nm colloidal gold particles coupled to protein A (CMC) were added to samples as fiducial markers. For correlative light and microscopy work, holey carbon grids were used and samples were stained with FM5–95 and/or DAPI directly before sample application to the grid and subsequent plunge freezing.

*Electron microscopy*. Cryo-electron tomography was performed on a Tecnai 20 FEG operated at 200 kV and a Titan Krios operated at 300 keV (FEI Company). Images were recorded using Explore 3D software on a 2 × 2 k^2^ camera mounted behind a GIF energy filter (Gatan) operated at a slit width of 20 eV. Over 100 cryo-electron tomograms of streptomycetes were recorded with 2° tilt steps between −60° and +60° at a defocus of −15 μm, at magnifications between × 1,850 (6.6 nm pixels size) and × 8,000 (1.64 nm pixel size).

*Cryo-correlative light and electron microscopy*. Plunge-frozen EM grids containing fluorescently labelled *Streptomyces* were imaged using a fluorescence microscope equipped with a THMS600 or CMS196 cryo-light microscope stage (Linkam), in conjunction with a Leitz DMRB (Leica), with a × 100 dry objective with a working distance of 4.7 mm and a numerical aperture of 0.75. Digital images were recorded with a Leica DFC350FX CCD camera. Following cryo-fLM imaging, sample grids were stored in liquid nitrogen until they were used for cryo-EM. Over 50 cryo-CLEM images were recorded.

### Image analysis and visualization

Tomographic tilt series were processed using IMOD version 4.5 (ref. 40). Projection images were pre-processed by hot pixel removal and rough alignment by cross-correlation. Final alignment was done using fiducial gold markers. The tomograms were obtained using a weighted back-projection or a simultaneous iterative reconstruction technique. Cryo-electron tomograms were Fourier filtered and denoized with a nonlinear anisotropic diffusion^41^ to enhance the visibility of structures. For 3D surface rendering, the tomographic volumes were imported into AMIRA (FEI) for further processing and representation. Cryo-CLEM overlays were produced by manual overlay (Adobe Photoshop) of different magnification TEM and fLM images using grid bars, streptomycetes, holey carbon support film and fiducial gold markers for alignment.

### Data availability

The data that support the findings of this study are available within the article or from the corresponding author upon request.

## Additional information

**How to cite this article:** Celler, K. *et al.* Cross-membranes orchestrate compartmentalization and morphogenesis in *Streptomyces*. *Nat. Commun.* 7:11836 doi: 10.1038/ncomms11836 (2016).

## Supplementary Material

Supplementary Information Supplementary Figures 1-9, Supplementary Tables 1-2

Supplementary Movie 1 *Streptomyces* membrane-filled tip. Three-dimensional reconstruction of a *Streptomyces albus* membrane-filled tip. A light ribosome- and macromolecular complex-free area can be seen forming a ribbon behind the tip. For scale, refer to Figure 1c-f.

Supplementary Movie 2 Tomogram and reconstruction of data in Figure 2a showing a *Streptomyces* hypha.

Supplementary Movie 3 Tomogram and reconstruction of data in Figure 2b showing a *Streptomyces* hypha containing small membranes.

Supplementary Movie 4 Tomogram and reconstruction of data in Figure 2c showing a *Streptomyces* hypha containing constricting membranes.

Supplementary Movie 5 Tomogram and reconstruction of data in Figure 2d showing a *Streptomyces* hypha containing full cross-membranes. The start of cross-wall formation is evident within the membranes.

Supplementary Movie 6 Fluorescence recovery after photobleaching on an *S. coelicolor* hypha constitutively expressing eGFP and stained with FM5-95 for membranes. Fluorescence recovery after photobleaching (FRAP) on a *S. coelicolor* strain constitutively expressing eGFP and stained with FM5-95 for membranes demonstrates cytosol permeability of membranes, evident when bleached GFP molecules spread through the membrane.

Supplementary Movie 7 Fluorescence recovery after photobleaching on a *S. coelicolor* hypha constitutively expressing eGFP and stained with FM5-95 for membranes. Fluorescence recovery after photobleaching (FRAP) on *S. coelicolor* constitutively expressing eGFP and stained with FM5-95 for membranes demonstrates cytosol impermeability of membranes, evident when the membrane prevents the flow of bleached GFP molecules.

Supplementary Movie 8 Fluorescence recovery after photobleaching on an *S. coelicolor* hypha constitutively expressing eGFP and stained with FM5-95 for membranes and WGA-Alexa 633 for cell wall. Fluorescence recovery after photobleaching (FRAP) on an *S. coelicolor* strain constitutively expressing eGFP and stained with FM5-95 for membranes and WGA-Alex 633 for cell wall demonstrates that cross-membranes not containing cross-walls can be both cytosol permeable (green arrows) and impermeable (red arrows). The bleached areas are denoted by white arrows.
