# Supplementary material for: Cross-membranes orchestrate compartmentalization and morphogenesis in Streptomyces
Source: Nat Commun. 2016 Jun 13;7:ncomms11836. doi: 10.1038/ncomms11836 (PMC4909990; doi:10.1038/ncomms11836)
Supplement: Supplementary Information — Supplementary Figures 1-9, Supplementary Tables 1-2 [file ncomms11836-s1.pdf]

SUPPLEMENTARY INFORMATION

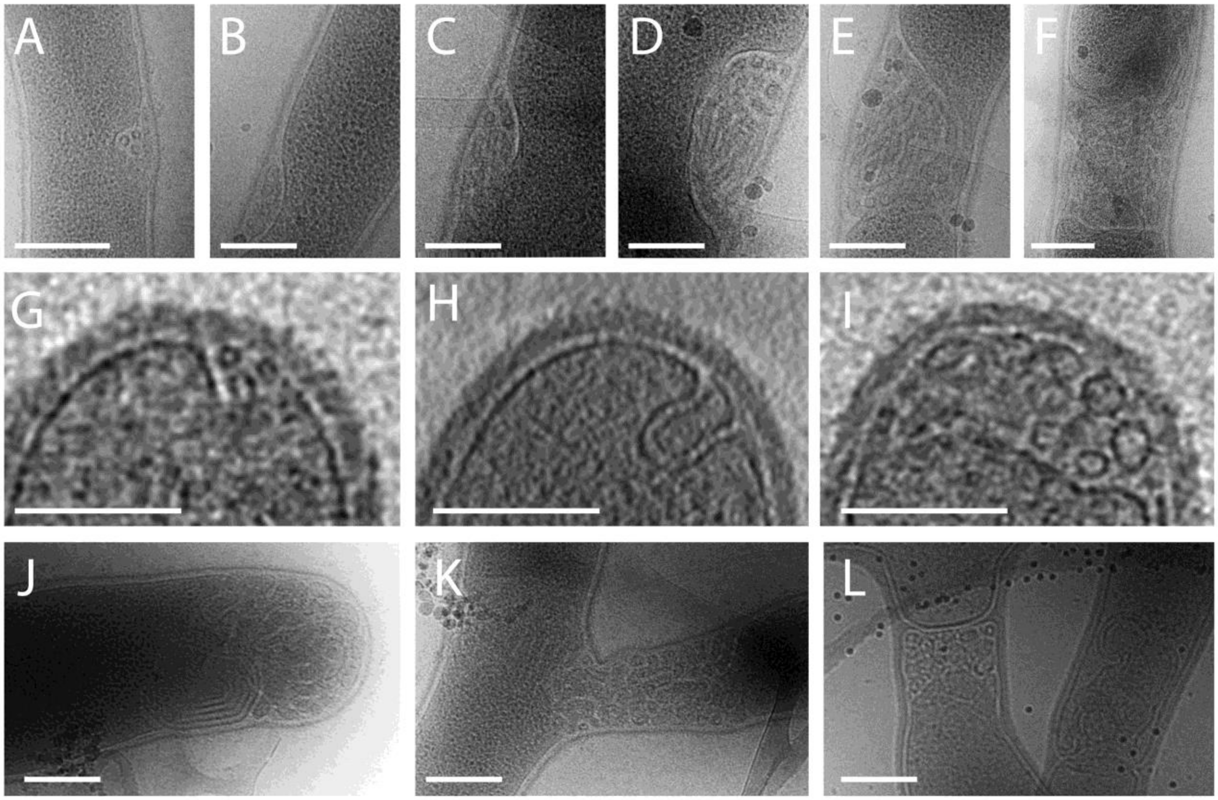

Supplementary Figure 1. Diverse intracellular membrane structures are found in *Streptomyces* sp. Membranes localize at the sides of hyphae in small or large blurbs (A-D) or to tips (G-J). Cell delimiting cross-membranes can also be found, with membrane tubes creating large intracellular structures (EFK). In some instances, cell wall can be found within these membrane structures (L).

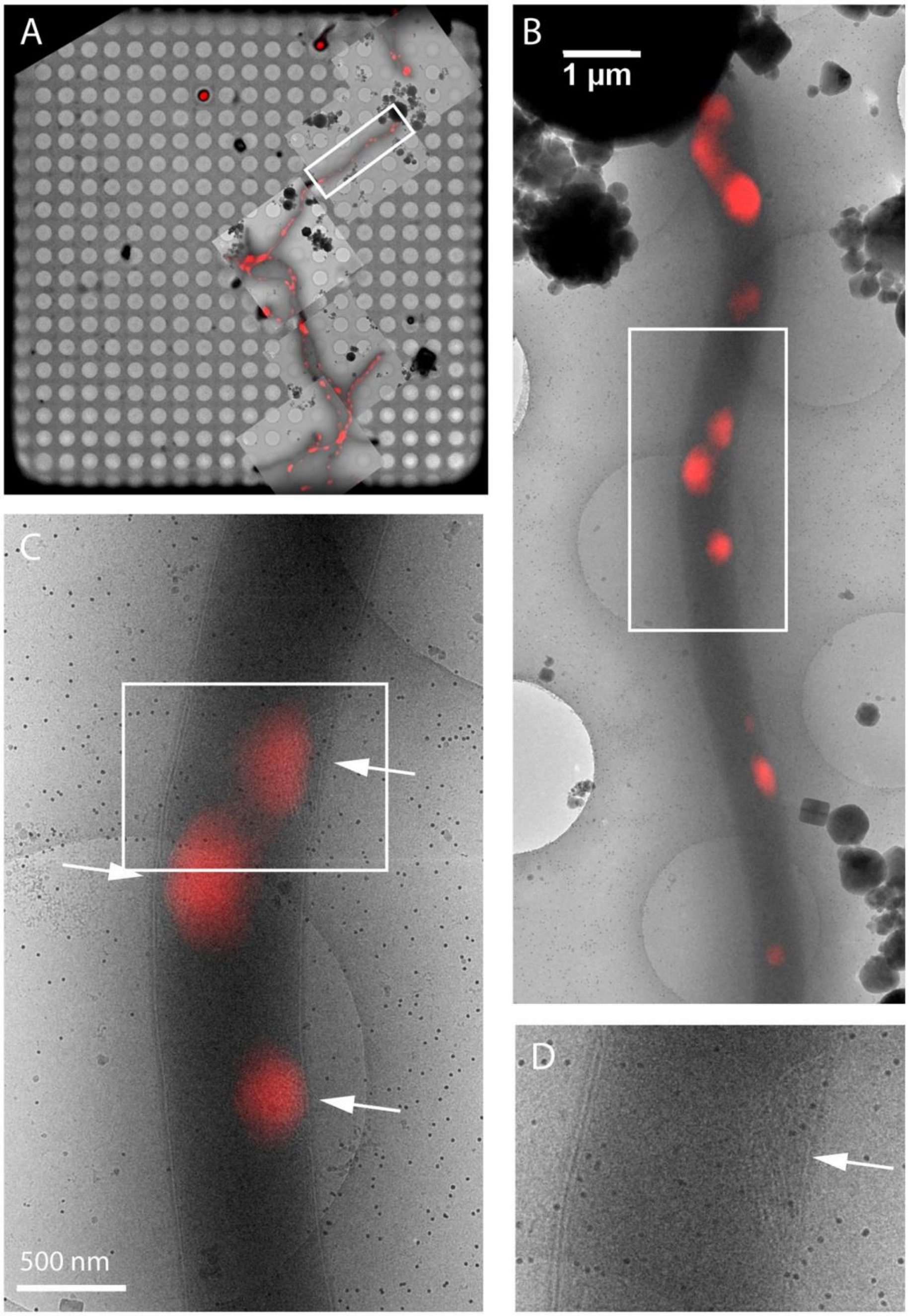

Supplementary Figure 2. Membrane assemblies can be found in *Streptomyces coelicolor.* Membrane assemblies are evident in a young vegetative hypha of *S. coelicolor* stained with FM5-95 and plunge frozen on a TEM grid (A, overlay of LM and fLM images). A TEM image of the boxed area is provided in B. Further magnification (C, D) reveals membrane assemblies at sites of FM5-95 staining.

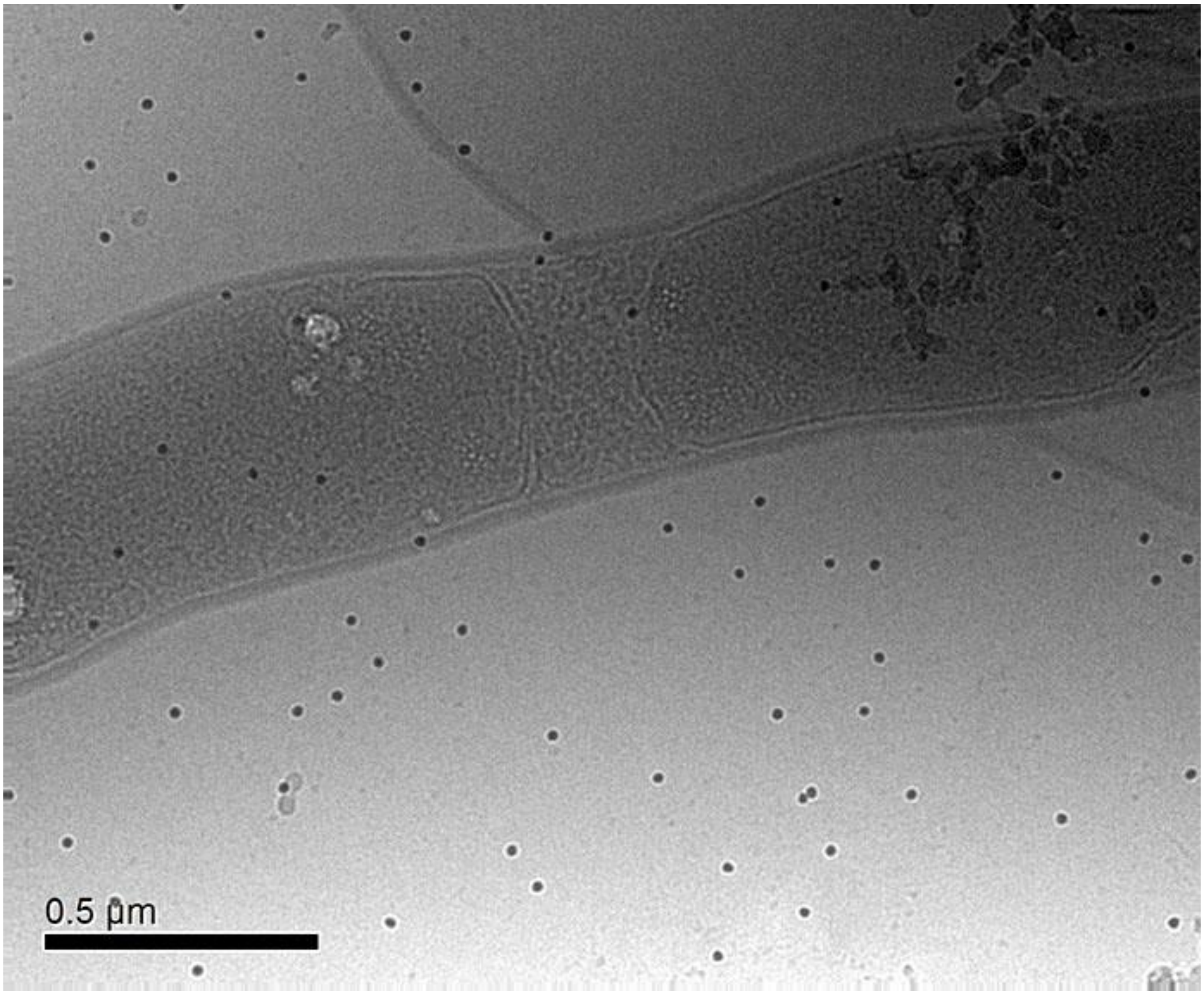

Supplementary Figure 3. Cryo-EM of membrane structures in an *S. coelicolor ftsZ*-deletion strain.

Cross-membranes in the ftrsZ deletion strain appear indistinguishable to those in wild type S. coelicolor

A

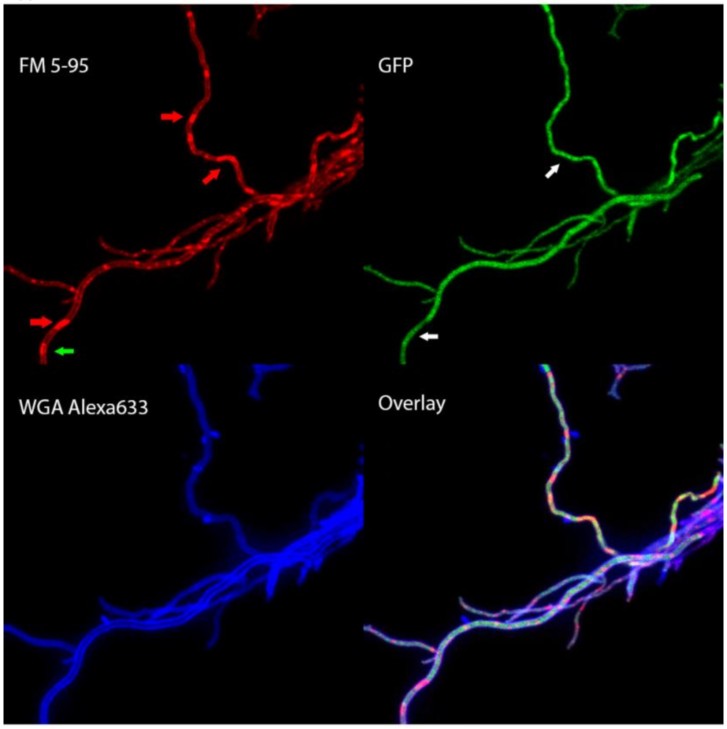

B

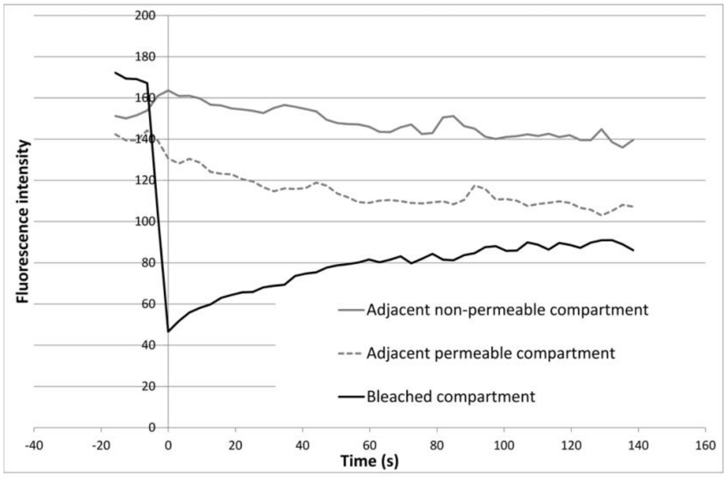

Supplementary Figure 4. FRAP analysis of GFP distribution over cross-membranes in absence of cross-walls. To confirm that impermeable membranes do not contain cell wall material, the FRAP analysis on the *S. coelicolor* strain constitutively expressing eGFP and stained for cross-membranes with FM5-95 (red) was repeated with cell walls stained with far-red dye WGA Alexa633 (false-coloured blue). Hyphal segments found between cross-membranes containing no cell wall material were bleached (white arrows), demonstrating that cross-membranes lacking cross-walls can be both permeable (green arrows) and impermeable (red arrows, see Supplementary Movie 8). In the graph the fluorescent recovery of the central bleach spot is shown together with the corresponding profiles of the two immediately adjacent compartments.

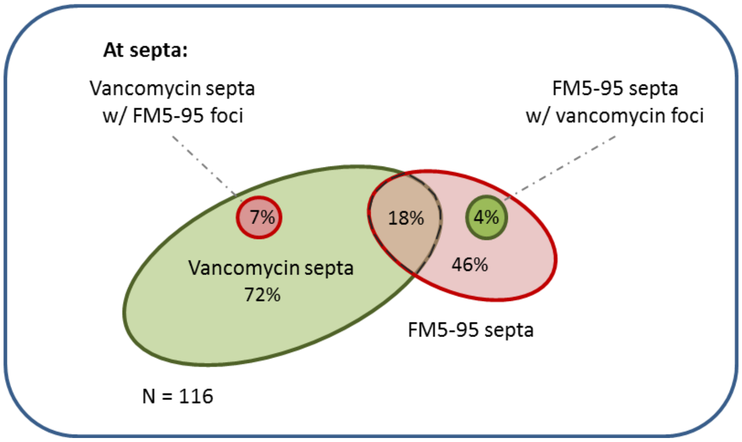

Supplementary Figure 5. Venn diagram of septal FM5-95 and BODIPY-colocalizations.

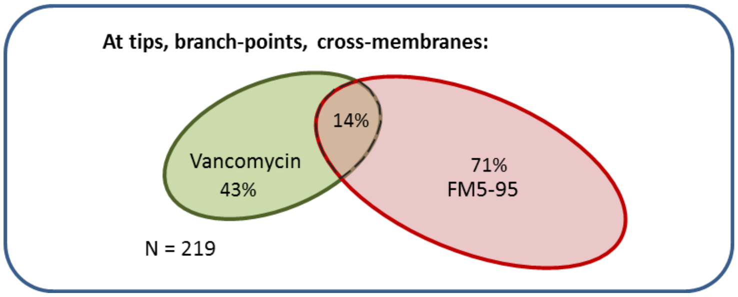

Supplementary Figure 6. Venn diagram of non-septal (tip, branch-point, and cross-membrane) FM5-95 and BODIPY-colocalizations.

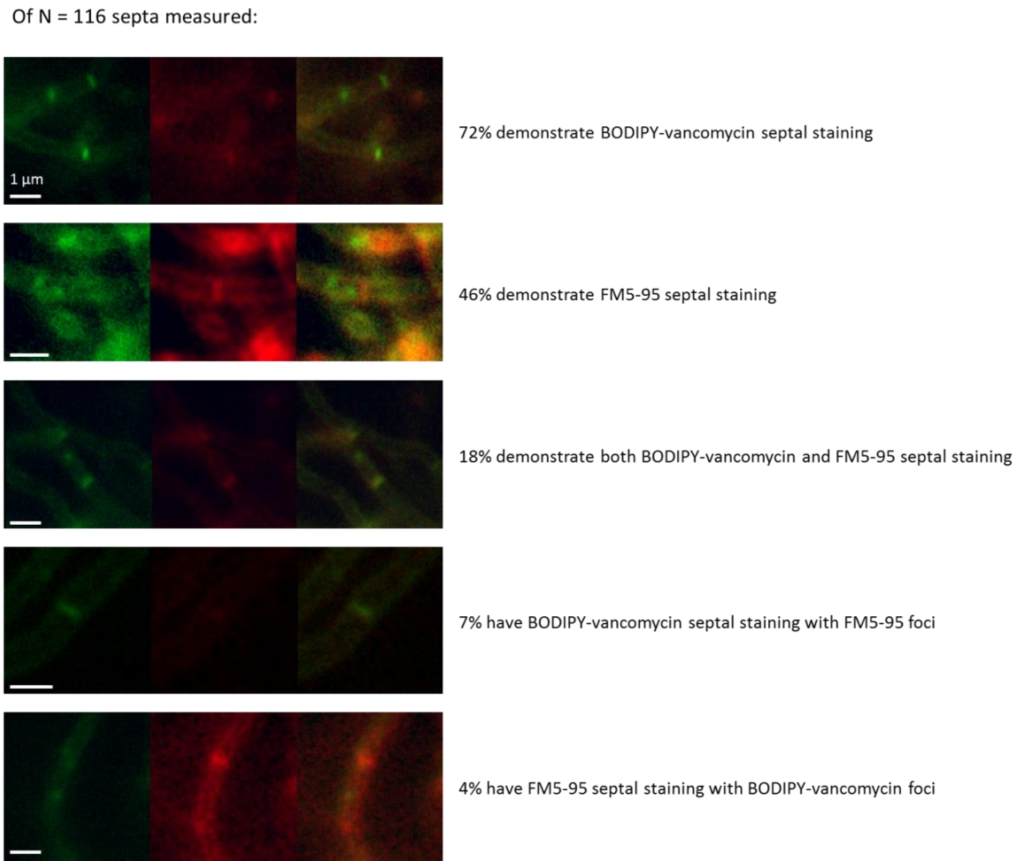

Supplementary Figure 7. Summary of septal colocalization scenarios observed during staining of *Streptomyces* vegetative hyphae with BODIPY-vancomycin (green) and FM5-95 (red).

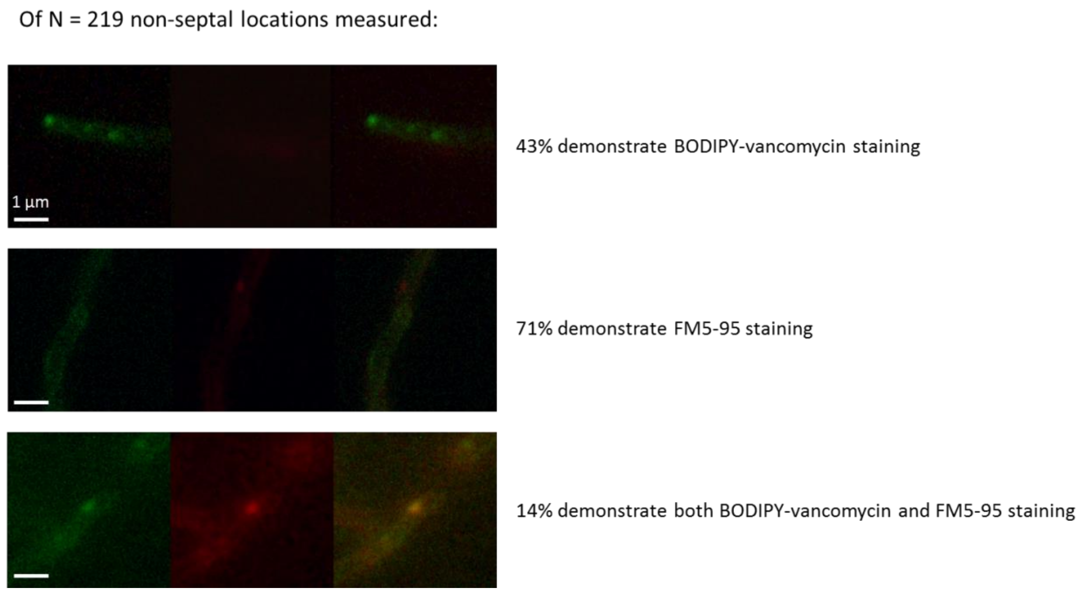

Supplementary Figure 8. **Summary of non-septal colocalization scenarios observed during staining of *Streptomyces* vegetative hyphae with BIDOPIY-vancomycin (green) and FM5-95 (red).**

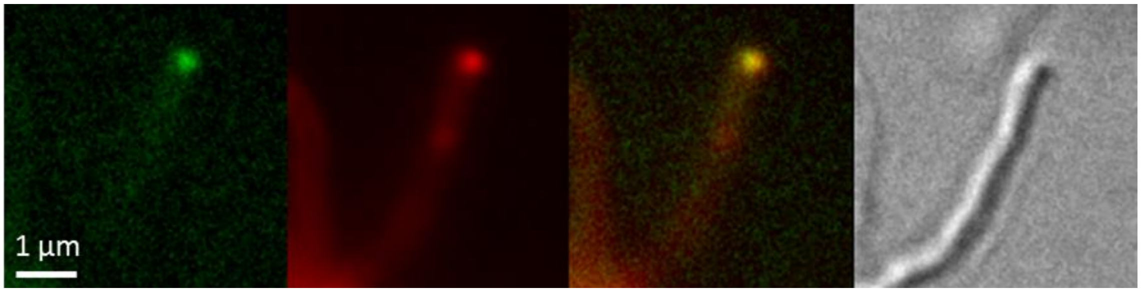

Supplementary Figure 9. Colocalization of DivIVA-GFP (green) and FM5-95 (red) foci in a *Streptomyces* hyphal tip. Overlay of the two channels demonstrates full colocalization at the tip (yellow). The light image is shown in the right-most panel.

Supplementary Table 1. Quantification of colocalization of FM5-95 and BODIPY-vancomycin staining in *Streptomyces coelicolor* vegetative hyphae (N=335). Venn Diagrams present the data for septal localizations (Supplementary Fig. 5) and non-septal (tip, branch-point, and large cross-membrane) localizations (Supplementary Fig. 6). Supplementary Fig. 7 shows examples of all of the localization scenarios presented in the Venn diagrams.

	FM5-95 staining at septum	FM5-95 foci	No FM5-95 staining	
vancomycin staining at septum	21	8	54	→at forming septa
vancomycin foci	5	31	63	→at reforming walls
no vancomycin staining	28	125		
	{ septa }	{ cross-membranes }		

Localizations counted: 335

Supplementary Table 2. Quantification of colocalization of FM5-95 and DivIVA-GFP foci in *Streptomyces coelicolor* vegetative hyphae (N=188). Of all locations analyzed, 45% contained only DivIVA, 39% contained only FM5-95, and the remaining 16% showed colocalization of DivIVA and FM5-95 foci (see Supplementary Fig. 9), with only one exception, in which a DivIVA focus colocalized with a FM5-95 stained septum.

	FM5-95 staining at septum	FM5-95 foci	No FM5-95 staining	
DivIVA staining at septum	0	1	6	→at growing septa
DivIVA foci	1	29	78	→at growing tips
No DivIVA localization	0	73		
	{ septa }	{ cross-membranes }		

Localizations counted: 188
